# A Practical Treatise on Nervous Exhaustion

**Published:** 1889-03

**Authors:** 


A PRACTICAL TREATISE ON NERVOUS EXHAUSTION. By the late G. M. Beard, M. D. Edited by Dr. A. D. Rockwell. Pages 254. Price, $2.75. E. B. Treat & Co., New York city.

This volume is a reprint of Dr. Beard's work upon neurasthenia or nervous exhaustion. The subject is one we may all well study thoroughly, for the disease, in its many manifestations, is very often seen. The American is perhaps more prone to this nervous exhaustion than any other person.

